# CROSS-CULTURAL ADAPTATION AND VALIDATION OF THE ITCHING SEVERITY SCALE IN CHILDREN AND ADOLESCENTS WITH ATOPIC DERMATITIS

**DOI:** 10.1590/1984-0462/;2017;35;3;00016

**Published:** 2017-07-13

**Authors:** Dayanne Mota Veloso Bruscky, Ana Caroline Cavalcanti Dela Bianca Melo, Emanuel Sávio Cavalcanti Sarinho

**Affiliations:** a Universidade Federal de Pernambuco, Recife, PE, Brasil.

**Keywords:** Translating, Pruritus, Atopic eczema, Preschool child, Child, Adolescent

## Abstract

**Objective:**

To translate, adapt and validate the Itch Severity Scale to a Brazilian version (ISS-Ped) in order to measure the severity of pruritus in children and adolescents with atopic dermatitis.

**Methods:**

This is a methodological study of validation of an instrument following recommended protocols. The translated version was evaluated by a group of experts including one professional with experience in instrument validation, three English teachers, one linguistics teacher and seven allergists. After this, the scale was applied to 42 parents of children aged between 2 and 18 years old with atopic dermatitis, and 42 parents of children without pruritic diseases. Results were evaluated according to the severity of atopic dermatitis and disease control, and they were compared between groups with and without atopic dermatitis.

**Results:**

More than 90% of the questions were clear to the parents. The ISS-Ped showed a strong positive correlation with the severity of atopic dermatitis (Pearson: 0.74; *p*<0.001) and a good correlation with the control of dermatitis (point-biserial correlation coefficient: 0.65; *p*<0.001). The scale showed excellent internal consistency (Cronbach’s α: 0.96) and adequate test and retest agreement (95% confidence interval of intraclass correlation coefficient: 0.89-0.99; *p*<0.001).

**Conclusions:**

The ISS-Ped is a feasible, valid, reliable and satisfactorily equivalent. The translated scale was appropriate to assess the severity of itching in children and adolescents with eczema, allowing comparisons in the clinical practice and in the research setting.

## RESUMO

**Objetivo:**

Realizar tradução, adaptação e validação da *Itch Severity Scale* (ISS-Ped) para medir a gravidade de prurido em crianças e adolescentes com dermatite atópica.

**Métodos:**

Estudo metodológico de validação de instrumento, seguindo protocolos recomendados. Após a definição da versão traduzida, com participação de um profissional com experiência em validação de instrumentos, três professores de inglês, um professor de linguística e sete alergologistas, a Escala de Gravidade de Prurido (ISS-Ped) foi aplicada a 42 responsáveis por pacientes entre 2 e 18 anos de idade com dermatite atópica e a 42 responsáveis por indivíduos sem doença cutânea pruriginosa da mesma faixa etária. Resultados da escala foram comparados com a gravidade da dermatite atópica e o controle da doença, e entre os dois grupos.

**Resultados:**

A clareza das questões foi maior que 90%. A ISS-Ped mostrou forte correlação positiva com a gravidade da dermatite atópica (Pearson: 0,74; *p*<0,001) e boa correlação com o controle da doença (coeficiente de correlação ponto bisserial: 0,65; *p*<0,001), além de ótima consistência interna (α de Cronbach: 0,96) e adequada reprodutibilidade pela concordância do teste e reteste (coeficiente de correlação intraclasse variando de 0,89 a 0,99 com IC95% e *p*<0,001).

**Conclusões:**

A ISS-Ped apresentou-se viável, válida e confiável, sendo atingida equivalência satisfatória. A escala traduzida mostrou-se adequada para avaliar a gravidade do prurido em crianças e adolescentes com dermatite atópica, permitindo comparações na prática clínica e entre pesquisas de diferentes centros.

## INTRODUCTION

Atopic dermatitis is the most common chronic inflammatory and relapsing skin disease in childhood, characterized by cutaneous xerosis and significant pruritus.^1^ ^,^ ^2^ It affects 10% to 20% of children and 1% to 3% of adults worldwide. In Brazil, phase III of the International Study of Asthma and Allergies in Childhood (ISAAC) found an estimated prevalence of 13.1% in children aged 6 to 7 years and 8.4% in adolescents aged 13 to 14 years.^3^ ^,^ ^4^ It is a disease that negatively affects the patient’s health and quality of life, damages the quality of life of their family members, and imposes a high economic cost on families, especially in its most severe forms.^5^ ^,^ ^6^ The main complaints are: sleep and mood alteration, social exclusion, children missing classes, as well as sleep deprivation and parents’ exhaustion. Persistent pruritus, which causes sleep and concentration impairment, in addition to social stigmatization, is one of the main components of the disease that contributes to social, psychological and economic effects.^5^ ^,^ ^6^ ^,^ ^7^ ^,^ ^8^ ^,^ ^9^

Pruritus, an unpleasant sensation that causes skin itchiness, results from the activation of non-myelinated nerve fibers (CMi fibers) in the papillary dermis and in the epidermis. After activation, these fibers synapse with secondary neurons, whose projections are part of the contralateral spinothalamic tract and ascend to various brain areas involved in sensitivity, emotion, evaluation, reward and memory.^10^ ^,^ ^11^ The mechanisms that promote this activation are complex and still poorly understood within atopic dermatitis, since histamine released by cutaneous mast cells is only one of the mediators. In addition to these and inflammatory cells, other substances such as neuropeptides, cytokines (mainly IL-2 and IL-31), tachykinins, tryptase, prostaglandins, platelet activating factor, leukotrienes, serotonin and eosinophilic proteins have been implicated in the pathophysiology of pruritus.^2^ ^,^ ^12^ ^,^ ^13^

Currently, the most widely used instrument to assess pruritus is the visual analogue scale. However, although it is a valid instrument, it has a great limitation in that it only evaluates the intensity of the symptom and not how the symptom interferes in the life of the affected patient. Moreover, because it is completely subjective and dependent on personal interpretation, the measurement of pruritus with this type of scale is individual, and makes comparisons difficult between individuals and groups, mainly with different ethnicities, social and cultural conditions.^14^ ^,^ ^15^ ^,^ ^16^ The Itch Severity Scale (ISS) was developed and validated from the Yosipovitch questionnaire to quantify the severity of pruritus. It was validated in English for patients aged 18 to 70 years with psoriasis.^15^ The Spanish version was validated for use in children and adolescents with atopic dermatitis, and was composed of six questions and scores ranging from 0 to 18 points.^17^ However, there is no gold standard instrument to measure pruritus yet, and it is widely recommended that at least two instruments be used to reliably assess the intensity of the symptom in clinical trials.^18^

In order for an instrument to be used in a population other than the one for which it was developed and validated, translation, cultural adaptation, and an analysis of the conceptual, item, semantic and operational equivalence are recommended. Additionally, tests that evaluate the measurement properties of these instruments in the new version, such as internal consistency, reproducibility, validity and viability are suggested.^19^ ^,^ ^20^ ^,^ ^21^ This study aimed to translate and adapt the ISS to Portuguese and Brazilian culture and to test its psychometric properties in children and adolescents with atopic dermatitis.

## METHOD

This is a study of translation, cross-cultural adaptation, and validation of a questionnaire that assesses the severity of pruritus in children and adolescents with atopic dermatitis. The study was conducted between May and December of 2014 in the Allergy and Immunology and Dermatology and Pediatrics outpatient clinics of the Hospital das Clínicas of Universidade Federal de Pernambuco (HC-UFPE), by a single and properly trained interviewer, in a one-on-one interview, and with duration of 10 to 15 minutes. On the day of collection, data from the patient’s medical history about the severity of his or her atopic dermatitis, as well as his or her need for corticosteroids in the consultation, were noted.

The following participated in the study: a committee of experts (allergists, English teachers, a linguistics professor and a researcher with experience in instrument validation); the caregivers of patients aged 2 to 18 years with a clinical diagnosis of atopic dermatitis^22^ ^,^ ^23^ and who received care at the HC-UFPE Allergy and Immunology clinic; the guardians of patients of the same age group without a pruritic cutaneous condition, and who received care at a general pediatric outpatient clinic in the same institution.

The scale consists of seven questions and its scores range from 0 (no pruritus) to 21 points (most severe pruritus), with a clinically important difference of two points. It has questions about description, frequency, intensity, and extent of pruritus, and how it affects sleep, mood, and sexual activity or desire. Each question is worth zero to one point, and the scale score is obtained by the sum of all questions multiplied by a correction factor (x3).

The validation process of the instrument was performed according to the steps described below^20^ ^,^ ^21^ ^,^ ^24^ and started after the authors of the original scale gave permission:

1. Assessment of conceptual and item equivalence, performed by an allergist, with the objective of assessing the relevance of the instrument and its items for atopic dermatitis.
2. Assessment of semantic equivalence, including two independent translations from the English language by two professionals fluent in the language (Translation 1 and 2 - T1 and T2, respectively); A synthesis of the translations was made by an observer who is fluent in English (T12); Two T12 back-translations were done, independently, by two native English teachers fluent in Portuguese (RT1 and RT2) and a synthesis of the back translations was completed (RT12); A verification of the equivalence was done by the committee of experts; They compared T12, RT12 and the original versions (each evaluation had the following options to choose from: “Exactly the same meaning”, “Almost the same meaning” and “Different meaning”); A pilot study with the translated version, in which the clarity and degree of comprehension were evaluated according to whether each item was “not clear at all”, “ a little unclear”, “clear”, “very clear” or “completely clear” for each question.
3. Operational equivalence was performed by means of an evaluation of the format, order and layout of the questions and the manner the instrument was applied.
4. Initial psychometric properties were analyzed as follows:

- viability was assessed by the percentage of responses given by the patients;
- convergent validity, compared to the severity of the atopic dermatitis measured by the Scoring of atopic dermatitis (SCORAD) and with or without the disease controlled;
- for the divergent validity, scale values were compared between patients with the disease and children without pruritic cutaneous condition;
- reproducibility was examined by retesting the 7 to 14-day interval in ten patients at random;
- the internal consistency of the instrument was calculated.

The data was organized into tables in duplicate in Microsoft^®^ Excel^®^ 2013 (Microsoft Corporation, Redmond, Washington, USA). The variables “age”, “sex”, “severity of atopic dermatitis” and “disease activity” were analyzed. Additionally, the frequency of understanding of the questions was analyzed under “clarity of the items”, considering the sum of “Clear”, “Very clear” and “Completely clear” to be greater than 90% for each question as adequate. Semantic equivalence was deemed adequate by the sum of “Exactly the same meaning” and “Almost the same meaning” being greater than 80% in the form filled out by the committee of experts.

The inferential analysis was done with SPSS 18.0 software (SPSS Inc., Chicago, USA) in order to perform the following tests. Values of p <0.05 were considered significant:

- promax rotation exploratory factor analysis, after a test of the construct in relation to the adequacy of the sample using the Kaiser-Meyer-Olkin index (KMO); values above 0.80 were considered sufficient; The Bartlett sphericity test was used in order to verify if the variables are correlated; the value of p up to 0.05 was considered adequate; ^25^
- feasibility, measured by the frequency of responses of the selected patients;
- comparison of the average scale between individuals with atopic dermatitis and those without pruritic cutaneous disease using Student’s t-test;
- analysis of the correlation between ISS-Ped and the severity of atopic dermatitis (measured by SCORAD),^26^ using the Pearson correlation index; the complete SCORAD was used and was filled out by a medical assistant during the consultation; it included an extensive evaluation of lesions, of intensity (erythema, infiltration/papule, crusting, excoriation, lichenification and xerosis) and of the subjective symptoms (pruritus and interference in sleep using an analogic visual scale);
- evaluation of the correlation between the translated scale and the disease activity (controlled or uncontrolled), demonstrated by the need for the use of topical corticosteroids in the consultation on the day of the interview, using the analysis of the point-biserial correlation coefficient ;
- reliability, assessed through internal consistency using Cronbach’s α coefficient (adequate when greater than 0.70) and through reproducibility and stability, measured by an intraclass correlation between the test and retest.

This study was approved by the Research Ethics Committee of the Federal University of Pernambuco (UFPE) (report nº 610.734 and Certificate of Presentation for Ethical Assessment nº 16628714.2.0000.5208). All participants aged 12 to 18 years and their caregivers signed the Informed Consent.

## RESULTS

The ISS was considered appropriate to measure the severity of pruritus in patients with atopic dermatitis, according to the researchers. All items were adequate, except for item 6, which was related to the influence of the symptom on the patient’s desire and sexual activity. It was withdrawn from the final scale for the pediatric population, resulting in a change in the scale score from 0 (no pruritus) to 18 (peak pruritus).

Semantic equivalence was performed through the evaluation of the original versions, T12 and RT12 by the committee of experts, which was composed of three English teachers, one professor with experience in instrument validation, one linguistics professor and seven allergists from several regions of Brazil (one from the North, three from the Northeast, two from the Southeast and one from the Midwest). In this process, all of the questions were considered appropriate, with an equivalence of 100.0% for all items, except question 3, which obtained 91.7%. The original version and the ISS-Ped are shown in Table 1. There were modifications by adding synonyms in questions 1, 2, 3 and 4; some terms were changed in question 5 (“depression” for “depressed”, “more agitation” for “more agitated”, “anxiousness” for “anxious”), in addition to the exchange of a word in question 7 (“on account” exchanged for “because”).

**Table 1:**
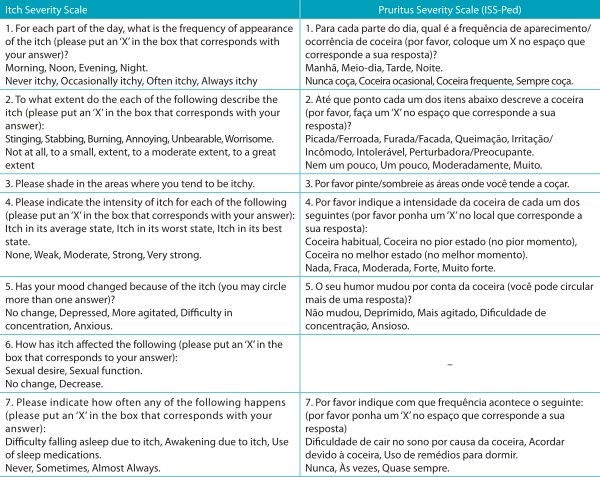
Itch Severity Scale and the final version of the Pruritus Severity Scale.

In the pilot study, the translated version was applied to patients with atopic dermatitis and those without pruritic disease (of whom composed the final group). The individuals understood the translated version of the scale, with the sum of “Clear”, “Very clear” and “Completely clear” above 90.0% for each question, as presented in Table 2. All questions were answered by the parents or caregivers of the children from all of the age groups. The children sometimes could or could not help with the answers.

**Table 2:**
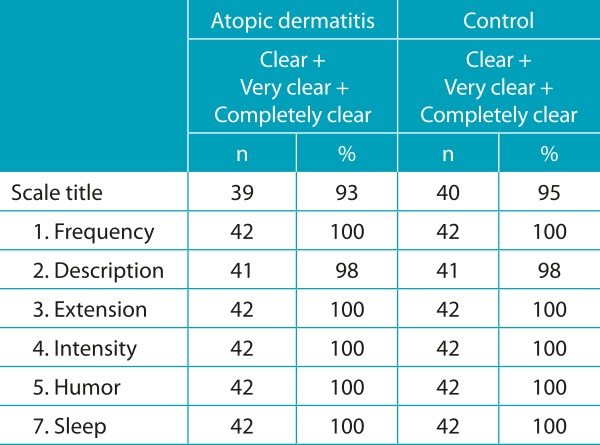
The atopic dermatitis and control groups’ assessment of the clarity of the items, by means of the sum of the answers “Clear”, “Very clear” and “Completely clear”.

A total of 43 patients aged between 2 and 18 years was recruited to participate in the study, and 42 (98%) caregivers answered the questionnaire, totaling 7 patients per each item of the scale. The average age of the patients was 7 ± 4 years, with 23 (55%) males. Regarding the severity of atopic dermatitis, 26 (62%) patients had the mild form of the disease and 10 (24%) had the severe form. Eczema was not controlled in 22 (52%) of the evaluated patients.

Forty-two caregivers of children who do not have pruritic cutaneous disease were also recruited, and all (100%) responded to the ISS-Ped. The average age was 7 ± 3.5 years, with 23 (55%) male patients. There was no statistical difference between the group with atopic dermatitis and the control group in relation to age and sex.

The same layout, order and format of the questions from the original version was maintained. However, due to the population’s difficulty in reading and understanding the scale, it was necessary to change the mode of its application. It changed from a self-applied scale to a face-to-face interview. In the initial phase of the pilot study, the caregivers did not read the questions, chose answers at random, and failed to answer all of the questions. When confirming if the items were clear, they demonstrated that they had not read the questions. Instruction was increased so that the caregivers of the minors could answer questions about the patient.

The average from the ISS-Ped in patients with atopic dermatitis was 8.0 ± 4.7 and, in patients without pruritic disease, 0.7 ± 1.1. This difference was statistically significant (p <0.001).

In the measurement equivalence process, the scale was considered viable when in the form of an interview, since 98% of the caregivers of children and adolescents with atopic eczema and 100% of the caregivers of children without pruritic skin disease, responded to the whole scale.

The analysis of the main components of the scale, after confirming satisfactory sample appropriateness (KMO = 0.85) and a correlation of the variables (Bartlett’s sphericity test with p <0.001), showed that a single component explained 69.1% of the variance, verifying the one-dimensionality of the translated version of the ISS.

**Figure 1:**
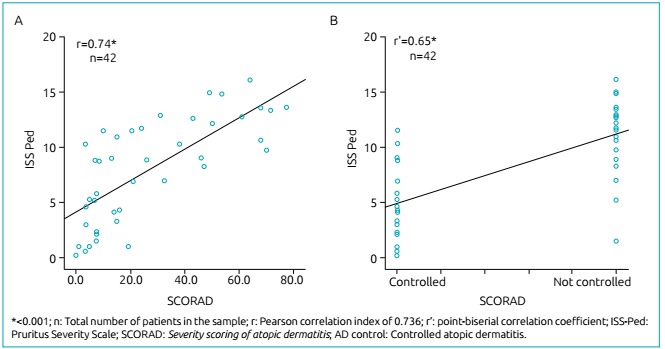
Correlation between the Pruritus Severity Scale and the Severity scoring of atopic dermatitis (A) and the atopic dermatitis control (B).

The ISS-Ped had a strong positive correlation with the severity of the SCORAD-measured dermatitis and a strong correlation with the uncontrolled disease for the studied sample, which were both statistically significant. The correlation data is shown in the scatter plot contained in Figure 1.

The internal consistency analysis of the scale was adequate, with a Cronbach’s α coefficient of 0.96. The reproducibility was also good, with a consistency measurement, through the intra-class correlation coefficient, of 0.97 (95% CI: 0.89-0.99) for the total scale (p <0.001). The evaluation of the reproducibility for each item had an intraclass correlation coefficient greater than 0.80 and p <0.05 for all questions. The retest was performed at intervals of 7 or 14 days (70% of the time with an interval of 7 days) by the same interviewer.

Table 3 shows the characteristics of the sample and the measurement properties of the ISS-Ped translated version.

**Table 3:**
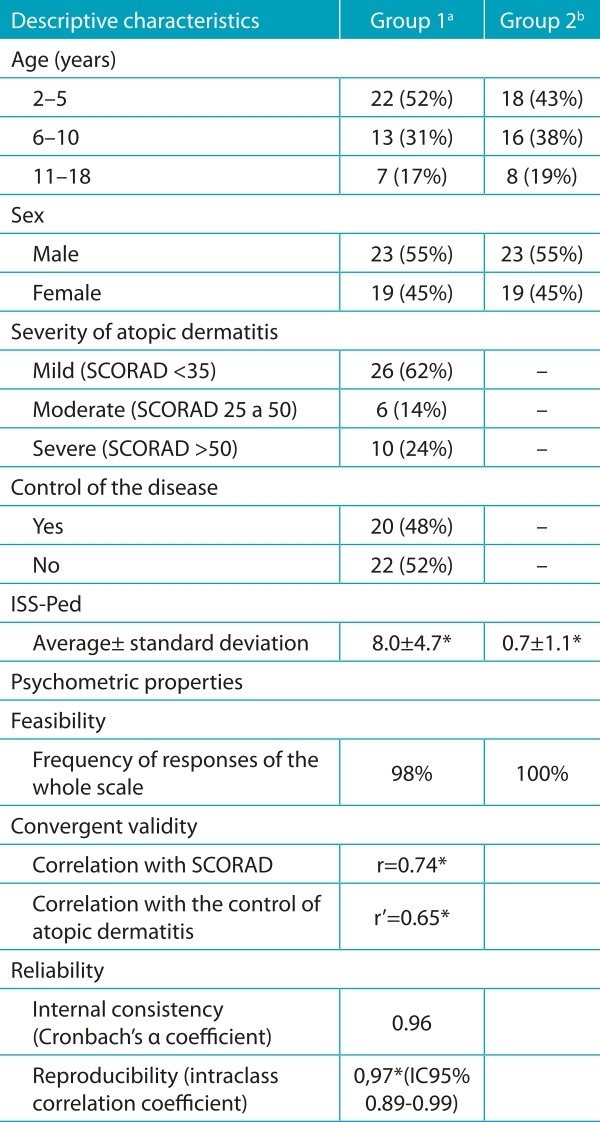
Description of the sample and the psychometric properties of the Pruritus Severity Scale for children and adolescents with atopic dermatitis.

apatients with atopic dermatitis; bchildren without pruritic skin disease; *p<0.001. SCORAD: Severity scoring of atopic dermatitis; ISS-Ped: Pruritus Severity Scale; r: Pearson correlation index; r’: point-biserial correlation coefficient; CI: confidence interval.

## DISCUSSION

This study demonstrated that the translated version of the ISS, the Pruritus Severity Scale (ISS-Ped), is valid for evaluating the severity of pruritus in children and adolescents with atopic dermatitis, and had measurement properties compatible with the original scale.

Satisfactory conceptual, item, semantic and operational equivalence were obtained. The removal of the question about interference in sexuality had already occurred in the Spanish version of the instrument,^17^ and is a necessary adjustment for the pediatric population. The stages of translation, back-translation and evaluation by the committee of experts were completed without major difficulties. Since the original scale is very clear, it allowed for the transference of concepts from the original instrument to the new version. Allergists from various regions of Brazil made up part of the committee in order to try to minimize the use of local terms that could not be understood in all regions of Brazil. The change in the *modus operandi* of the interview was necessary and did not interfere in the measurement properties of the scale, maintaining its effectiveness.^21^ ^,^ ^27^ The “interview” method has the advantage of decreasing the frequency of blank responses and avoiding misunderstandings in interpretation. However, the disadvantage is that it requires a trained interviewer.^25^

Both the caregivers of atopic dermatitis patients and the caregivers of children without pruritic disease satisfactorily understood the “interview” version of the scale. There was an expected and significant difference between the severity of the pruritus assessed in these two groups: the group without pruritic disease had an average close to zero, indicating no pruritus and corroborating the validity of the scale.

The ISS-Ped is feasible, based on the high frequency of participation and the population’s response to all items, just like in the original version and the Spanish version.^15^ ^,^ ^17^

The convergent validity of the scale was performed through a comparison with SCORAD, which measures the severity of atopic dermatitis, and the control of the disease. The theoretical model demonstrates that patients with a disease that is more severe and uncontrolled, and who, as a result, need therapy, have more severe skin inflammation and itching.^1^ ^,^ ^2^ ^,^ ^6^ ^,^ ^7^ ^,^ ^9^ The ISS-Ped showed a good correlation with the severity of the atopic dermatitis (Pearson> 0.7), confirming that the greater the severity of the disease, the greater the severity of pruritus, since there is more cutaneous involvement and a more important pruriginous symptom. When ISS-Ped was compared with control of the disease, there was also adequate and statistically significant correspondence. The original version had a moderate correlation with the “physical health” (r = 0.483) and “mental health” (r = 0.492) modules of the RAND 36 Health Status Inventory, and a good correlation with the Dermatology Quality of Life Index (DLQI) (r= 0.628),^15^ while the Spanish version had a good correlation with the Pediatric Quality of Life Index in Dermatology (r = 0.69).^17^

In the exploratory factor analysis, the one-dimensionality of the translated version of the scale was observed, showing only the severity of pruritus. In the construction of the original scale, two dimensions were found in the initial stage. In ISS, after the removal of items, five of the total items dealt with severity, and two of them with temporality.^15^ In this study and in the Spanish version, the strength of the second item was insufficient, but the scale, which aims to measure the severity of the symptom, was not impaired.

The reliability of the instrument was demonstrated by the high Cronbach’s α coefficient (0.96), which was comparable to the original version (0.80) and the Spanish one (0.84), proving that the different items of the scale are correlated and measure the same construct. The reproducibility was also satisfactory. It was evaluated by the correlation between the test and retest and included data that was already observed in the original version (Pearson correlation coefficient equal to 0.95 and intraclass correlation coefficient of 0.95)^15^ and in the previous validation of the scale.^17^ It is believed that, because it allows for fewer skipped responses, the operational modification has been responsible for increasing the internal consistency of the instrument.

Some limitations should be considered in extrapolating the results obtained. Even with the attempt to reduce the influence of colloquial language, and the revision of the scale by doctors from different regions of Brazil, the instrument was applied in a sample of patients from Pernambuco and cultural and socioeconomic differences should be considered. It is also necessary to emphasize that children aged above 10 years present a greater possibility of describing their pain, and offer a more reliable description of the symptom. However, studies with pain and asthma show a good correlation between the responses of the patients and their caregivers,^28^ ^,^ ^29^ permitting such use.

In conclusion, it is considered that the Pruritus Severity Scale will be of great use in clinical practice to evaluate the efficacy of pruritus treatment and for future research on the evaluation of the treatment of atopic dermatitis in our population. Additionally, it allows for comparisons to be made between studies performed in different centers that use the instrument more effectively.
